# Health and Social Care Staff Awareness of Menopausal Symptoms in Adults With Intellectual Disability: Results From a Survey

**DOI:** 10.1192/bjo.2024.451

**Published:** 2024-08-01

**Authors:** Jack Wilson, Niall O'Kane, Bernice Knight, Angelica Lindsey-Clark, Jessdeep Rai

**Affiliations:** 1 Islington Learning Disabilities Partnership, London, United Kingdom; 2 Whittington Hospital, London, United Kingdom

## Abstract

**Aims:**

Menopausal symptoms often go unrecognised in individuals with intellectual disability (ID). There is growing societal awareness of the impact of menopause on mental health, yet this has not been replicated in the ID population. In light of this, we wanted to establish the current levels of knowledge, confidence and skills of staff working in a specialist community intellectual disability service (CIDS). The findings from the survey may help identify ways of improving awareness of menopausal symptoms with individuals with ID.

**Methods:**

We performed a cross sectional survey of staff views and practice in relation to considering and discussing menopausal symptoms with individuals with ID. The survey was anonymous, and conducted on Microsoft Forms. A mixture of quantitative and qualitative data was captured. A QR code linking to the survey was disseminated to the whole team (60 staff) via email and in-person staff meetings.

**Results:**

There was 50% (30/60) responses to the staff survey. The majority of respondents worked in either health (16/30) or social care (12/30). Two thirds of respondents either agreed or strongly agreed (20/30) that discussing menopausal symptoms was part of their role. 57% of respondents (17/30) felt confident discussing menopausal symptoms with service users, while 20% (6/30) felt neutral and 23% did not feel confident. 90% (27/30) of respondents either agreed or strongly agreed that they would benefit from teaching and training in the effects of menopause in our service users. Thematic analysis of the free text responses revealed that staff wanted to understand treatments available for menopause as well as improved easy read material explaining menopausal symptoms to individuals with ID.

**Conclusion:**

Our survey revealed a spectrum of confidence levels in discussing menopausal symptoms with service users, and a large appetite for further training and resources to aid these conversations. In light of the results from this survey, a Quality Improvement (QI) project has been initiated. Once QI change ideas have been tested, a repeat survey will be completed to compare staff views and confidence in this area and in this way measure the effectiveness of those changes.

